# Evaluating the Impact of Diabetes on Hernia Management by Exploring Pathologies and Comorbidities in High-Risk Patients

**DOI:** 10.7759/cureus.86329

**Published:** 2025-06-19

**Authors:** Mostafa Ahmed Abdellah Ahmed, Zeeshan Hussain, Noor Ul Ain Rashid, Seemi Tanvir, M Khaliq

**Affiliations:** 1 General Surgery, Frimley Health NHS Foundation Trust, Frimley, GBR; 2 Underwater and Hyperbaric Medicine, Pakistan Navy Station Shifa Hospital, Karachi, PAK; 3 Hyperbaric and Diving Medicine, Armed Forces Aero Medical Center, Dhahran, SAU; 4 General Surgery, Akhtar Saeed Trust hospital, Lahore, PAK; 5 Pathology, Margalla Institute of Health Sciences, Rawalpindi, PAK; 6 Pathology, University of Health Sciences, Lahore, PAK

**Keywords:** diabetes mellitus, hernia repair, postoperative outcomes, surgical site infection (ssi), wound healing

## Abstract

Background: Diabetes mellitus is a recognized risk factor for impaired wound healing and greater postoperative complications. Thus, this study was conducted to assess the effect of diabetes on outcomes of hernia management in high-risk patients, including wound healing, recurrence, and postoperative infections.

Methods: This retrospective observational study was carried out between March and August 2024 and features 120 recruited patients who underwent elective hernia repair. The patients were stratified into two groups: group A (diabetics; n=60) and group B (non-diabetics; n=60). Age, BMI, comorbidities, and the American Society of Anesthesiologists (ASA) score were recorded as preoperative data. Operative time, wound healing duration, incidence of surgical site infections (SSIs), and recurrence over six months were analyzed during intraoperative and postoperative periods from surgical records. Statistical analysis was carried out with SPSS Statistics version 26.0 (IBM Corp., Armonk, NY, USA), using the chi-square test and independent t-test with a p-value < 0.05 as statistically significant.

Results: Diabetics had significantly longer operative times (p = 0.001). Patients in group A (with prolonged drainage time) had longer wound healing (17.2 ± 3.4 vs. 12.5 ± 2.7 days, p < 0.001), higher incidence of SSI (21.7% vs. 6.7%, p = 0.015), and longer drain retention (p < 0.001). Diabetics also had significantly longer hospital stays (6.3 vs. 4.5 days; p < 0.001). Diabetic recurrence was more than in non-diabetics at six months (11.7% vs. 3.3%), despite a p-value of 0.085. Diabetic patients also expressed trends towards increased formation of seroma and chronic pain.

Conclusion: Diabetic patients requiring hernia repair are at higher risk for delayed healing of wounds, surgical site infection, and longer hospitalization.

## Introduction

Hernia repair is the most common general surgical procedure worldwide and, with few exceptions, is associated with good outcomes [1]. However, recovery and complication rates after hernia repair are influenced by comorbid conditions, particularly diabetes mellitus. Diabetes is a long-term metabolic disorder characterized by hyperglycemia resulting from defects in the secretion or action of insulin or both [2]. Its systemic impact includes impaired immune function, microvascular and macrovascular complications, and altered inflammatory responses, all important in surgical outcomes [3].

Diabetes remains unique among patients undergoing hernia repair. Impaired wound healing associated with poor glycemic control is due to reduced collagen synthesis, decreased leukocyte activity, and deficient tissue perfusion [4]. Diabetic patients are subsequently more at risk for the development of surgical site infections (SSIs), extended recovery times, and hernia recurrence [5]. Other diabetes-associated factors, such as obesity, poor nutritional status, and higher American Society of Anesthesiologists (ASA) scores, complicate perioperative management as well [6].

Although the risks associated with diabetes in hernia surgery are recognized, its effects on specific outcome measures after elective hernia surgery have not been fully defined, particularly in patients at elevated risk [7]. There are a few studies that systematically evaluate and compare wound healing, SSI rates, hospital stay duration, and recurrence rates between diabetic and non-diabetic cohorts. These associations are critical to tailor perioperative care, improve glycemic control, and mitigate risks.

This study aims to fill this knowledge gap by prospectively examining the effects of diabetes on hernia surgery outcomes. It provides evidence-based insights into wound healing time, infection rates, and recurrence within a defined follow-up period to support clinical decision-making in diabetic patients undergoing hernia repair.

## Materials and methods

This retrospective observational study was conducted to evaluate how diabetes mellitus may affect the results of an elective hernia repair, particularly by examining wound healing, possible recurrence, and postoperative infections. The research was conducted at a tertiary care hospital (Shaikh Zayed Hospital, Lahore, PAK) between March and August 2024. The sample size was determined using OpenEpi version 3.0.0 (OpenEpi, Atlanta, GA, USA). A total of 120 patients who underwent elective hernia repair were included in the study and were divided into two groups, namely diabetics (group A, n = 60) and non-diabetics (group B, n = 60). Each participant gave a consent form for the use of their clinical data in this study. Individuals enrolled in the study were selected through convenience sampling from hospital records, meeting the study’s criteria, and had undergone elective hernia surgery during the study period.

This study was approved by the Institutional Review Board of Shaikh Zayed Hospital (approval no. SURG31-23) and was conducted per the Declaration of Helsinki. Patients with emergency hernia repairs, weakened immune systems, chronic steroid use, or those with prior hernia repairs within the last year were excluded from the study. Age, BMI, comorbidities, and ASA physical status scores were noted before the procedure. Operative time and drain placement were taken as intraoperative variables. As an observational study design, no interventions were applied, nor were any variables manipulated. All data analysis was done with SPSS Statistics version 26.0 (IBM Corp., Armonk, NY, USA). Continuous variables are shown as mean ± standard deviation and were analyzed with independent t-tests. The percentages of categorical variables were calculated and compared using either the chi-square test or Fisher’s exact test (p-value <0.05).

## Results

A total of 120 patients undergoing hernia repair were divided into two groups. Group A comprised diabetics (n =60) and group B comprised non-diabetics (n = 60). Baseline demographic variables of age, sex, and BMI did not statistically differ, indicating successful group matching. Surgical times were longer and the ASA grades higher, in the diabetic cohort. Delayed healing and infections were more often postoperative wound-related complications for diabetics (p < 0.05).While recurrence rates among group A patients during the six month follow-up were also elevated, the difference did not approach statistical significance (Table 1).

**Table 1:** Baseline and operative characteristics Data were evaluated using independent t-tests and chi-square tests. ASA: American Society of Anesthesiologists

Variable	Group A (diabetics)	Group B (non-diabetics)	Test	Test value	p-value
Age (mean ± SD)	58.2 ± 9.5	53.6 ± 10.2	t-test	t = 2.58	0.012 *
Gender (M/F)	41/19	43/17	Chi-square	χ² = 0.17	0.69
BMI (kg/m² ± SD)	28.7 ± 3.6	27.4 ± 3.3	t-test	t = 2.01	0.048 *
ASA class III–IV (%)	33 (55%)	18 (30%)	Chi-square	χ² = 7.42	0.006 *
Duration of surgery (min ± SD)	83.4 ± 11.2	76.5 ± 10.8	t-test	t = 3.38	0.001 *

No difference was seen in gender or baseline age distribution. Diabetics had higher ASA status, BMI, and a significantly longer operative times. There were significantly higher wound infections and delayed healing in diabetics. Drain removal and hospital stay duration were also longer. All results were statistically significant (Table 2).

**Table 2:** Wound healing outcomes Data were analyzed using independent t-tests and chi-square tests. SSI: Surgical site infection

Parameter	Group A (diabetics)	Group B (non-diabetics)	Test	Test value	p-value
Delayed wound healing (%)	16 (26.7%)	5 (8.3%)	Chi-square	χ² = 7.39	0.006 *
Surgical site infection (%)	13 (21.7%)	4 (6.7%)	Chi-square	χ² = 5.86	0.015 *
Drain removal time (days ± SD)	4.1 ± 1.2	2.8 ± 1.1	t-test	t = 5.08	<0.001 *
Hospital stay (days ± SD)	6.3 ± 2.1	4.5 ± 1.6	t-test	t = 4.08	<0.001 *

Diabetics also had higher recurrence and seroma. The two groups were similar in chronic postoperative pain. Trends suggest that longer-term outcomes were poorer in diabetics (Table 3). These findings highlight that diabetes has a significant impact on the postoperative outcomes. Therefore, the need for customized management strategies in high-risk populations is crucial.

**Table 3:** Recurrence and long-term complications Data were analyzed using chi-square and Fisher’s exact tests.

Parameter	Group A (diabetics)	Group B (non-diabetics)	Test	Test value	p-value
Hernia recurrence (6 months) (%)	7 (11.7%)	2 (3.3%)	Fisher's Exact	–	0.085
Seroma formation (%)	10 (16.7%)	4 (6.7%)	Chi-square	χ² = 2.89	0.087
Chronic pain (6 months) (%)	6 (10%)	3 (5%)	Fisher's Exact	–	0.48

## Discussion

This retrospective observational study analyzed the effects of diabetes mellitus on post-hernia repair outcomes such as wound healing, infections, and hernia recurrence. The outcomes revealed that diabetic patients have longer wound healing durations, hospital stays, and operative times, and more SSIs compared to patients without diabetes. Hernias recurred at higher rates in the diabetic group, but the difference was not significant. It was found that both seroma formation and chronic pain were also increased in the diabetic group [8]. A group of researchers also found that diabetes was an independent predictor of postoperative complications, including recurrence [9]. Some studies found statistically significant increases in hernia recurrence among diabetics, but our experience showed only a non-significant trend; this may be explained by a difference in either sample size or the duration over which our cases were followed up [10]. Observations are consistent with those of the past, i.e., prolonged hospital stay and increased operative time for diabetics; however, these represent the healthcare resource implications to care for diabetic surgical patients [11].

Several potentially pathophysiological mechanisms can explain the adverse outcomes seen in diabetic patients. The impairment of immune defenses, such as leukocyte chemotaxis and phagocytic activity associated with chronic hyperglycemia, raises the risk of infection [12,13]. Globally, microvascular disease stemming from diabetes is such that tissue perfusion and oxygenation necessary for wound repair are reduced [14]. Furthermore, disturbed fibroblast function and fibrogenesis with subsequent collagen synthesis alter the normal healing processes and lead to impaired closure of the wound and, subsequently, early hernia recurrence [15]. The study by Shanahan et al. shows that optimized perioperative care in diabetic patients undergoing hernia repair is important. Minimization of complications is possible with careful preoperative glycemic control, strict aseptic technique, and appropriate prophylactic antibiotic use [16]. Because of the increased risk profile, surgeons should be aware and do detailed counselling regarding probable delays in recovery and complications [17]. Early detection and management of wound complications should be followed after operations, and long-term sequela should be prevented. There is further potential for strategies to improve wound healing, including new therapies for this vulnerable population [18].

Limitations of this study are a moderate sample size that may have limited the extent to which significant differences in recurrence can be detected for hernia. The six-month follow-up period is adequate for early complications, but the recurrences or late complications might not be captured during this period. Moreover, systematic data collection in glycemic control parameters, which include HbA1c, may also have contributed to outcomes. Further research is needed using larger, multicenter cohorts with follow-up for longer periods, incorporating more detailed metabolic control metrics. Further exploration is critical in interventions to improve wound healing and lessen infection risk in diabetic patients having hernia repair.

## Conclusions

A significant effect is found with outcomes in terms of delayed wound healing, increased SSIs, longer operative time, and prolonged hospital stays after hernia repairs compromised by diabetes mellitus. Although there wasn't a statistically higher hernia recurrence in diabetics, there was a clear trend toward the need for increased clinical vigilance. These results highlight the need for patient-specific perioperative strategies and close postoperative surveillance to optimize surgical outcomes in diabetic patients. Due to the unique challenges associated with diabetes, there is a need to address diabetes recovery concerning optimizing recovery and reducing complication rates in a high-risk population.
